# Supplementary material for: Study of the Interaction of a Novel Semi-Synthetic Peptide with Model Lipid Membranes
Source: Membranes (Basel). 2020 Oct 19;10(10):294. doi: 10.3390/membranes10100294 (PMC7603383; doi:10.3390/membranes10100294)
Supplement: Supplementary file 1 [file membranes-10-00294-s001.pdf]

membranes

Article

Supplementary Materials: Study of the Interaction of a Novel Semi-Synthetic Peptide with Model Lipid Membranes

Lucia Sessa [1,2,*], Simona Concilio [1,2,*], Peter Walde [3], Tom Robinson [4], Petra S. Dittrich [5], Amalia Porta [1,2], Barbara Panunzi [6], Ugo Caruso [7], and Stefano Piotto [1,2]

[1] Department of Pharmacy, University of Salerno, 84084 Fisciano (SA), Italy; aporta@unisa.it (A.P); piotto@unisa.it (S.P)
[2] Research Centre for Biomaterials BIONAM, University of Salerno, Via Giovanni Paolo II, 84084 Fisciano (SA) 132, Italy
[3] Department of Materials, ETH Zürich, 8093 Zürich, Switzerland; peter.walde@mat.ethz.ch
[4] Department of Theory and Bio-Systems, Max Planck Institute of Colloids and Interfaces, D-14424 Potsdam, Germany; tom.robinson@mpikg.mpg.de
[5] Department of Biosystems Science and Engineering, ETH Zurich, 4058 Basel, Switzerland; petra.dittrich@bsse.ethz.ch
[6] Department of Agriculture, University of Napoli Federico II, 80055 Portici (NA), Italy; barbara.panunzi@unina.it
[7] Department of Chemical Sciences, University of Napoli Federico II, 80126 Napoli, Italy; ugo.caruso@unina.it
* Correspondence: lucsessa@unisa.it (L.S); sconcilio@unisa.it (S.C)

Received: 30 September 2020; Accepted: 15 October 2020; Published: date

Microfluidic Device Design

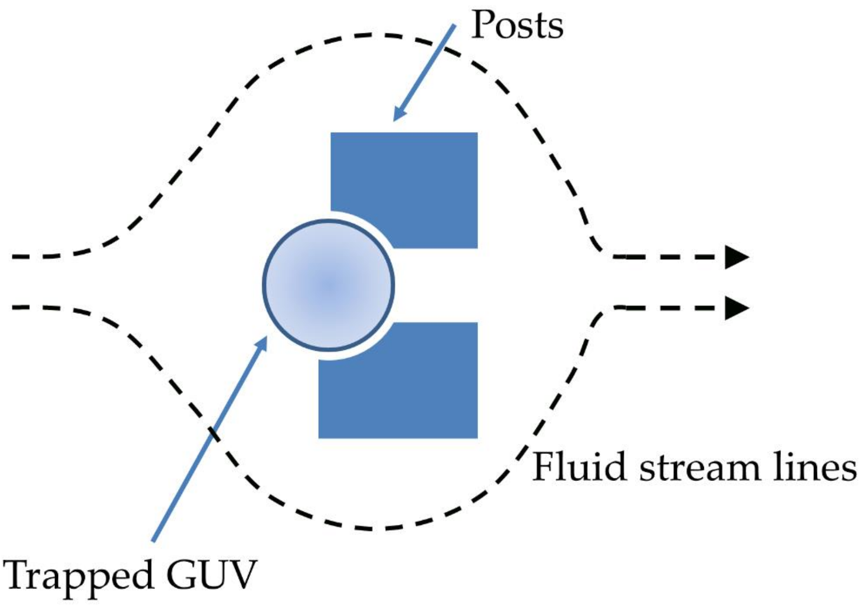

Figure S1. Scheme showing a trapped GUV isolated and the fluid flow around the trap. The chip and the valves are not shown in the figure, for details see ref [1].

Trapping of GUV

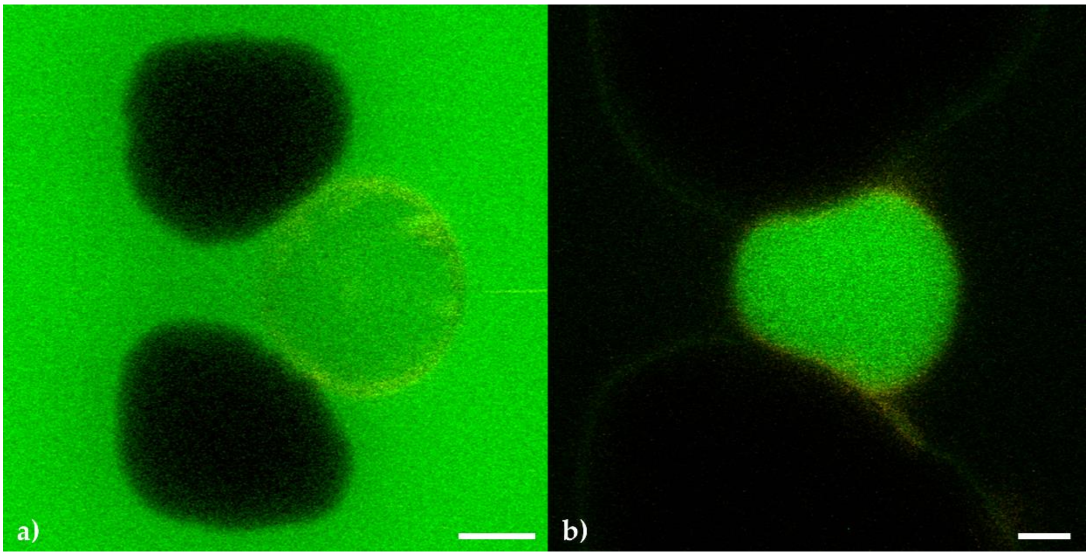

Figure S2. (**a**) Wide-field fluorescence image of a single GUV trapped hydrodynamically by the posts (black). Scale bar 5000 nm. The surrounding calcein solution (green) is diverted around the trap (**b**) The fluid was then exchanged for water without removing the calcein filled GUV. Scale bar 2000 nm.

Separation of Non-Entrapped HRPC from HRPC-Containing Vesicles

The non-entrapped enzyme molecules were separated from the enzyme-containing vesicles by size-exclusion chromatography using a 2 × 20 cm glass column filled with Sepharose 4B equilibrated with 10 mM MES buffer (pH = 5). 2 mL of the vesicle suspension was applied, and the separation was performed at a flow rate of 0.5 mL/min. Fractions of 1 mL were collected.

Absorption/turbidity measurements of each eluted fraction, performed with a spectrophotometer, indicate a good separation of the vesicles from the free enzyme. The optical density at 403 nm indicates two peaks, the first assigned to the turbid vesicles fraction, the second to free, non-entrapped HRPC with an absorption maximum at 403 nm due to the Soret band.

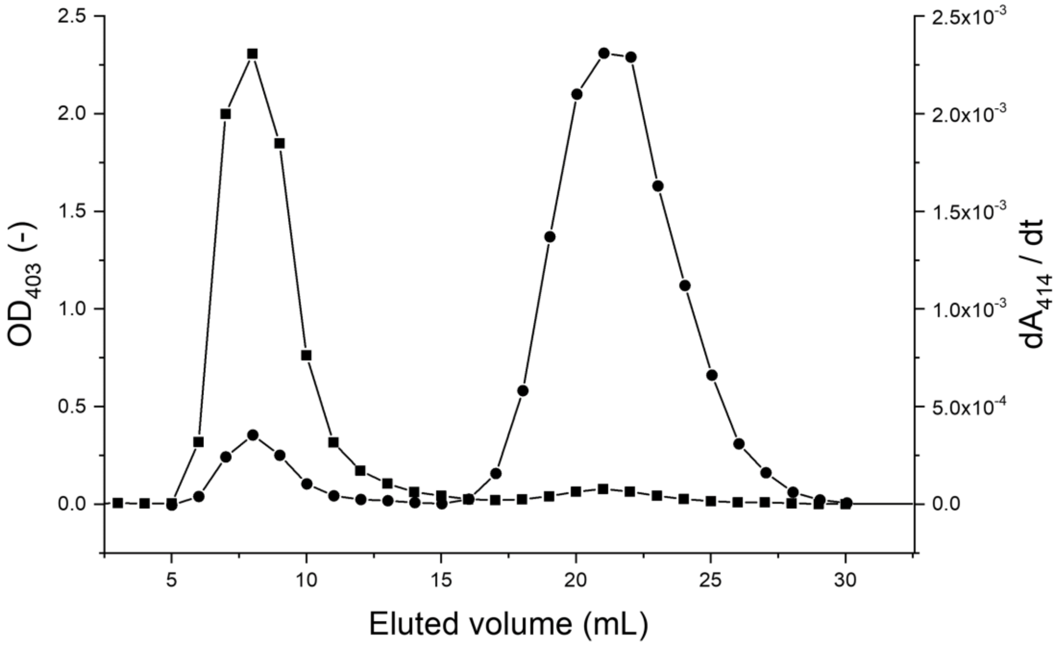

Figure S3. (–■–) OD_{403} originating from light scattering (turbidity) of the vesicles present in the fractions eluting up to an elution volume of about 12 mL, and (–•–) HRPC activity of the different fractions, measured with $ABTS^{2-}$ / H_2O_2 as substrates. In the case of the vesicle fractions, the activity was measured in the presence of 0.1 vol.% Triton X-100.

HRPC Activity Measurements

For measuring the activity of HRPC in solution, three stock solutions were freshly prepared:

1. An $ABTS^{2-}$ stock solution was prepared by dissolving a few mg of $ABTS^{2-}$ in 1 mL of 10 mM MES buffer solution (pH = 5). The exact concentration was calculated by UV absorption measurements (λ = 340 nm, ε_{340} = 36000 $M^{-1}\cdot cm^{-1}$) [2]. The solution was kept in the dark at room temperature and used within 8 h.
2. A H_2O_2 stock solution (4 mM) was prepared by appropriate dilution with MilliQ water of a 30 wt% aqueous H_2O_2 solution.
3. An enzyme stock solution (4 mg/mL) was prepared by dissolving HRPC in 10 mM MES buffer (pH = 5). From this concentrated solution, a diluted enzyme stock solution (1 μM) was prepared by using the same buffer solution. Finally, for each measurement, two fresh stock solutions (100 nM and 2 nM) were prepared in plastic reaction tubes.

The activity measurements were carried out in the following way: MES Buffer solution (10 mM, pH = 5.0) was added to a polypropylene (PP) reaction tube to reach the assay volume of 1 mL, $ABTS^{2-}$ (final concentration of 0.25 mM) and HRPC stock solution were mixed in the reaction tube. Immediately before the spectrophotometric analysis, H_2O_2 was added (final concentration of 80 μM). The mixture was transferred into a polystyrene (PS) cuvette (path length = 1 cm) and the change of the absorption spectrum of the reaction mixture was monitored as a function of time using a diode array spectrophotometer (Specord S600 from Analytik Jena). All measurements were repeated three times.

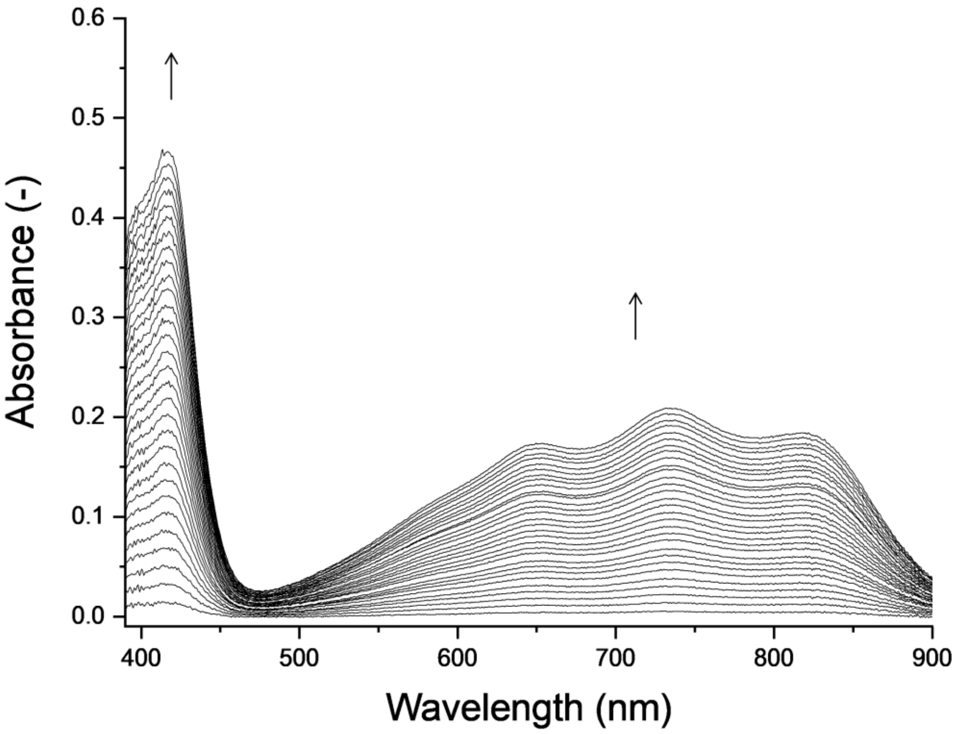

Figure S4. Changes of the absorption spectrum of the reaction solution as a function of reaction time, with $[ABTS^{2-}]_0$ = 0.25 mM, $[H_2O_2]_0$ = 80 μM and [HRP] = 150 pM at 25 °C (10 mM MES buffer pH = 5). The spectra were recorded at intervals of 10 sec immediately after the start of the reaction (up to 5 min).

Calibration Curves for HRPC Activity

Linear regression of the product absorbance at λ_{max} = 414 nm as a function of time allowed an easy determination of enzymatic activity, read as the slope of the linear fit (dA_{414} / dt).

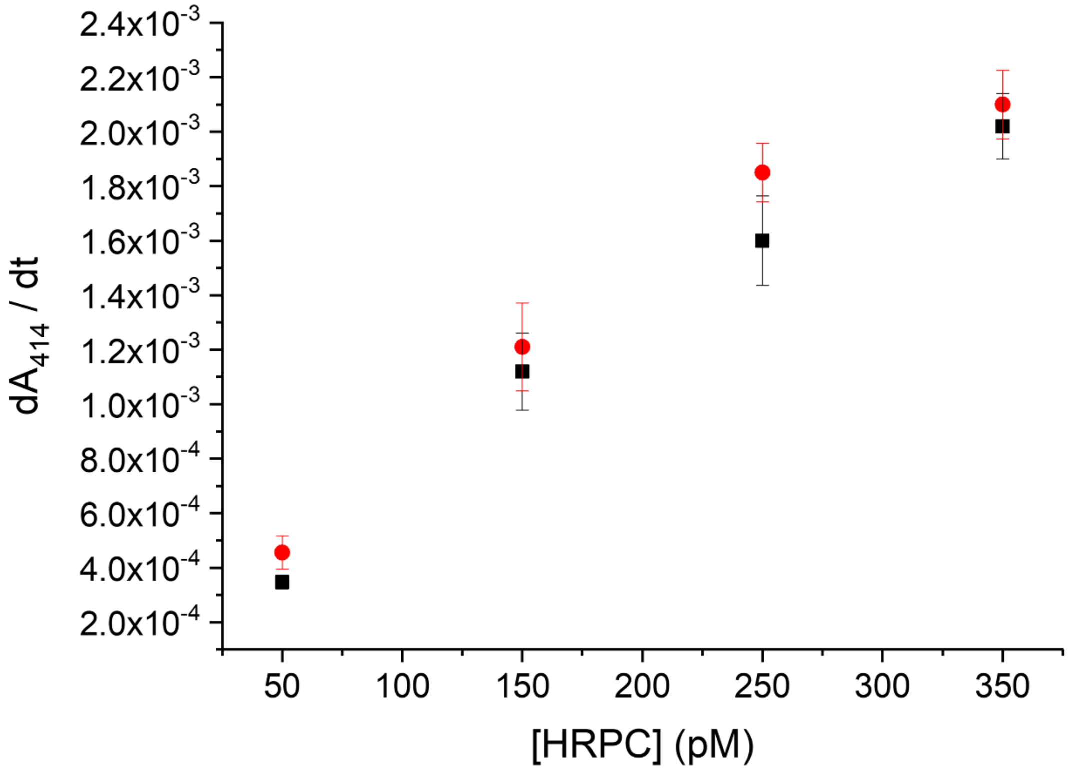

Figure S5. HRPC concentration dependency of the absorbance of the reaction solution at 414 nm measured during the first 5 min of enzymatic reaction without addition of detergent to the substrate mixture (–■–) and with addition of Triton X-100 (0.1 %) (–●–). Each data point shown is the average from three measurements using the same stock solutions. The standard deviation is indicated with error bars. Deviation from linearity was evident for HRPC concentration higher than 350 pM HRPC.

Membrane Perturbation Studies

The snapshot from the MD trajectory at time 0 of the modified peptide azoALY shows the insertion of the peptide in membrane with a tilt angle of 48° and with the hydrophobic portion (Ala-Leu-Tyr-Leu-Ala) immersed in the core membrane. In the lest snapshot at a simulation time of 50 ns, the azoALY peptide is still anchored in the membrane, with the azo amino acid intercalate in the core membrane.

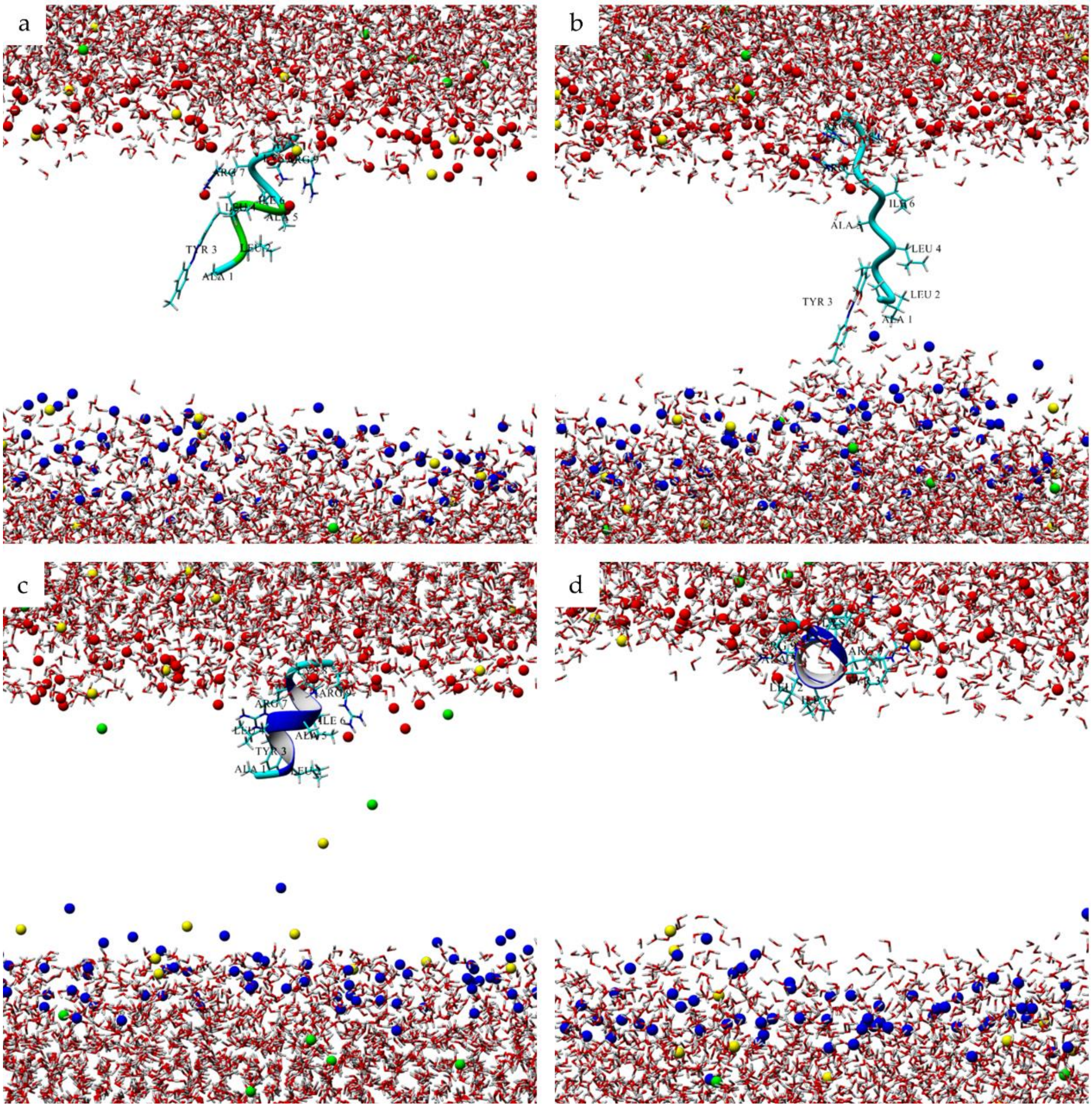

Figure S6. Snapshot of (**a**) the starting configuration of the azoALY/POPC-POPG system; (**b**) the last frame at 50 ns of simulation time of the azoALY/POPC-POPG system; (**c**) the starting configuration of the ALY/POPC-POPG system; (**d**) he last frame at 50 ns of simulation time of the ALY/POPC-POPG system. The water molecules around the membrane are represented in stick style and salt in ball style. The membrane shape can be traced from the positions of phosphorus atoms, showed in red and the blue for the two membrane leaflets.

In Figure S7, the SCDs for POPC/POPG phospholipid chains around 5 Å from the peptides ALY (in red) and azoALY (in blue) are shown. In Figure S7a, the unsaturated chain is shown, and in Figure S7b, the saturated one.

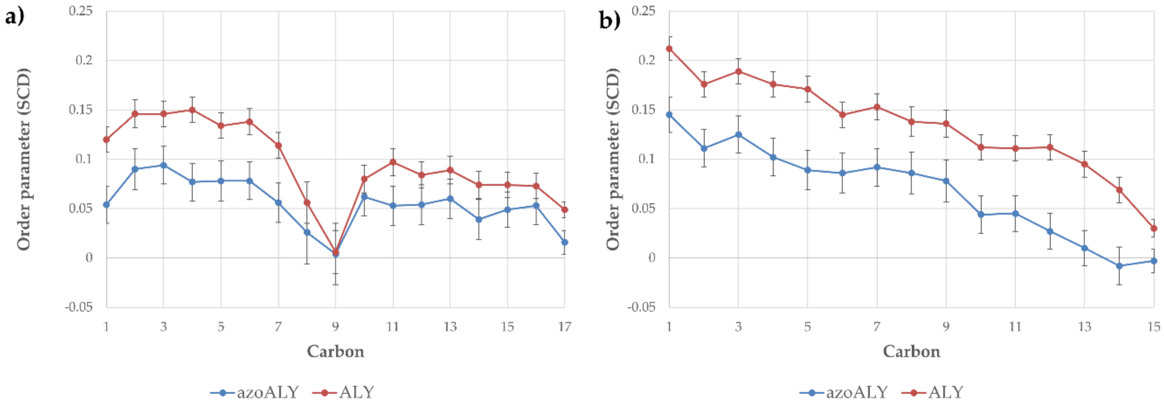

Figure S7. Order parameter SCD for (**a**) the unsaturated oleic and (**b**) the saturated palmitoyl acyl chains of phospholipids in POPC/POPG/peptide (azoALY, blue curve; Aly red curve).
Notes: On the Y-axis, the SCD is indicated; on the X-axis, the carbon atom position is reported, starting from the first (1) alpha carbon atom in the chains.

References

1. Robinson, T.; Kuhn, P.; Eyer, K.; Dittrich, P.S. Microfluidic trapping of giant unilamellar vesicles to study transport through a membrane pore. Biomicrofluidics **2013**, *7*, 44105–44105.
2. Childs, R.E. and W.G. Bardsley, The steady-state kinetics of peroxidase with 2, 2′-azino-di-(3-ethyl-benzthiazoline-6-sulphonic acid) as chromogen. *Biochem. J.* **1975**, *145*, 93–103.

Publisher's Note: MDPI stays neutral with regard to jurisdictional claims in published maps and institutional affiliations.

© 2020 by the authors. Submitted for possible open access publication under the terms and conditions of the Creative Commons Attribution (CC BY) license (http://creativecommons.org/licenses/by/4.0/).
